# Kidney angiopoietin-like protein 4 regulates fibrotic responses in diabetic kidney disease

**DOI:** 10.3389/fphar.2026.1803412

**Published:** 2026-04-15

**Authors:** Swayam Prakash Srivastava, Ajan Vishnu Arora, Mariam Hamed, Adesh Urval, Aashika Dupati, Shangyang Xia, Shota Yoshida, Ken Inoki

**Affiliations:** 1 Life Sciences Institute, University of Michigan, Ann Arbor, MI, United States; 2 Department of Molecular and Integrative Physiology, University of Michigan Medical School, Ann Arbor, MI, United States; 3 Department of Internal Medicine, University of Michigan Medical School, Ann Arbor, MI, United States

**Keywords:** c-GAS-STING pathway activation, defective lipid metabolism, diabetic kidney disease, inflammation, mitochondrial damage and dysfunction, podocyte-ANGPTL4, tubule-ANGPTL4

## Abstract

The angiopoietin-like (ANGPTL) proteins are now recognized as critical regulators of the harmful changes seen in diabetic kidney disease. Notably, a hyposialylated pro-proteinuric form of ANGPTL4 has been identified as a crucial contributor to fibrosis within diabetic kidneys. Under diabetic conditions, glomerular podocytes secrete this hyposialylated ANGPTL4, which plays a significant role in disrupting lipid metabolism, promoting inflammation, and driving fibrosis in both podocytes and adjacent tubules. This secreted ANGPTL4 interacts with tubular cells via Integrin-1 and DPP-4, causing mitochondrial damage that activates the c-GAS-STING pathway and exacerbates inflammation. Importantly, targeting renal ANGPTL4 with antisense oligonucleotides (ASOs) has shown promising protective effects against fibrosis in diabetic kidneys, paving the way for innovative therapeutic strategies to combat diabetic kidney disease and other fibrotic disorders.

## 1 Introduction

Diabetic kidney disease (DKD) has recently become a global health threat and serves as one of the leading causes of chronic kidney disease and end-stage renal disease (Thomas et al., 2015). As diabetes continues to rise in prevalence worldwide, there is strong reason to believe that additional molecular mechanisms are at play beyond those conventionally addressed (Cooper and Warren, 2019). Rather than emerging solely from hyperglycemia, DKD now seems to arise from the intersection of metabolic stress, microvascular damage, and inflammation (Cooper and Warren, 2019). Understanding these mechanisms is critical to developing new strategies to halt or intervene in kidney damage in diabetes (Srivastava et al., 2021a; Bhatia et al., 2023; Srivastava and Kanasaki, 2022; Bhatia and Srivastava, 2025; Srivastava, 2023). The current therapeutics for managing diabetes and the related kidney disease include sodium glucose cotransporter-2 inhibitors (SGLT2is), blood pressure-lowering agents such as angiotensin-converting enzyme (ACE) inhibitors, angiotensin receptor blockers (ARBs), N-acetyl-seryl-lysyl-proline (AcSDKP), non-steroidal mineralocorticoid receptor (MR) antagonists, and glucagon-like peptide-1 (GLP-1) receptor agonists (Aga et al., 2025; Agarwal et al., 2025; Bhandari et al., 2022; Srivastava et al., 2020a; Li et al., 2020a; Srivastava et al., 2020b; Nitta et al., 2016; Tuttle et al., 2022; Mazzieri et al., 2024; Watanabe et al., 2022). Effective treatment with SGLT2is or GLP1 receptor agonists (GLP-1 RAs) has been a key development in the treatment of DKD (Jensen et al., 2026; Shokravi et al., 2025; Michos et al., 2023). By decreasing proximal tubule glucose reabsorption, SGLT2is such as empagliflozin can restore tubuloglomerular feedback and lower intraglomerular pressure (Vallon and Thomson, 2017). Consequently, these medications show renoprotective effects, including improving glycemic control by reducing albuminuria, inflammation, and fibrosis (Vallon and Thomson, 2017; Speedtsberg and Tepel, 2023; Heerspink et al., 2016; Upadhyay, 2024). Even in individuals with normal glucose levels, trials, such as CREDENCE and DAPA-CKD, have shown an considerable slowdown of the progression of chronic kidney disease and a decreased risk of end-stage kidney diseases (Heerspink et al., 2021; Cherney and Verma, 2021; Chen et al., 2024). Notably, SGLT2is have also been shown to improve cardiovascular outcomes (Shokravi et al., 2025; Michos et al., 2023). Liraglutide and semaglutide are representative analogs of GLP-1 RAs that provide benefits by improving metabolic regulation, promoting weight loss, lowering blood pressure, and minimizing oxidative stress and systemic inflammation (Wong and Drucker, 2025; Abasheva et al., 2024). Additionally, new observations suggest that GLP-1 RAs may exert direct renoprotective effects by enhancing endothelial function and reducing glomerular damage (Wu et al., 2024; Chen et al., 2025; Jimenez et al., 2019; Mima et al., 2012; Hirata et al., 2009). GLP-1 RAs and SGLT2is provide cardio-renal protection that differs from renin-angiotensin system inhibition, providing an additional layer of DKD treatment (Colhoun et al., 2024; Cooper and van Raalte, 2025; Fioretto et al., 2016; Bae, 2025). Moreover, emerging therapies, including endothelin receptor antagonists (ERAs), dipeptidyl peptidase-4 (DPP-4) inhibitors (linagliptin), glycolysis inhibitors, fatty acid oxidation modulators, and non-steroidal glucocorticoid receptor (GR) agonists, have shown some renoprotective effects in the mouse model of DKD (Srivastava et al., 2020b; Mazzieri et al., 2024; Kanasaki et al., 2014; Srivastava et al., 2018; Srivastava et al., 2021b; Zhou et al., 2020; Srivastava et al., 2021c). However, it has not been well analyzed in large-scale, randomized clinical trials in human subjects (Kanasaki et al., 2014; Srivastava et al., 2018; Srivastava et al., 2021b; Srivastava et al., 2021c). However, most of the therapies have shown acute or mild side effects and have limited ability to improve the kidney structure and function and reverse the fibrogenic response in DKD.

The angiopoietin-like (ANGPTL) protein family has attracted significant attention as a group of secreted proteins that bridge metabolic pathways, vascular function, and immune modulation (Zhang et al., 2006; Morelli et al., 2020). The family comprises eight members (ANGPTL 1–8); all proteins share a conserved N-terminal coiled-coil domain and, except ANGPTL8, a C-terminal fibrinogen-like domain (Morelli et al., 2020). ANGPTLs differ from many classic angioproteins, as in general, ANGPTLs do not bind to Tie2 receptors but instead activate other cell-surface proteins, such as integrins and neuropilins (Santulli, 2014; Thorin et al., 2023). When first discovered, ANGPTLs were thought to promote angiogenesis mainly. Among all ANGPTL proteins, ANGPTL4 is of particular interest for its unique role in metabolic and vascular responses in the kidney and other organs. It is produced in many tissues, including adipose tissue, liver, muscle, heart, and in the podocytes and tubular epithelial cells of the kidney (Santulli, 2014; Li et al., 2024a; Carbone et al., 2018). ANGPTL4 levels are increased under metabolic stress, fasting, hypoxia, and in response to PPAR agonists; these elevated protein levels act as potent inhibitors of lipoprotein lipase (LPL), thereby enhancing triglyceride accumulation and distribution among tissues such as adipose, liver, heart, and kidney (Ruppert et al., 2020; Fernandez-Hernando and Suarez, 2020). Recent research has also shown its role in causing aberrant angiogenesis, vascular permeability dysfunction, vascular leakage, endothelial instability, inflammation, and tissue fibrosis, prompting renewed interest in ANGPTL4’s relevance to DKD and its potential as a biomarker or therapeutic target (Srivastava et al., 2025a). This review synthesizes findings from animal and human studies, emphasizing how the progression of DKD may be attributed to the effects of angiopoietin-like 4 (ANGPTL4) on metabolic signaling, vascular dysfunction, inflammatory, and fibrogenic pathways (Santulli, 2014; Li et al., 2024a; Carbone et al., 2018; Srivastava et al., 2024). This review critically discusses the potential of ANGPTL4 as a biomarker of DKD and explains mechanistically how does tubule-specific and podocyte-specific ANGPTL4 lead to lipid load, mitochondrial damage, inflammation and defective lipid metabolism, and accumulative effects accelerate the fibrogenesis process in diabetic kidney. Targeting kidney specific ANGPTL4 is a new and safe strategy in the management of fibrotic manifestations in DKD.

## 2 The ANGPTL family in kidney health and disease

The kidney produces multiple ANGPTL proteins, each contributing to renal homeostasis and pathology in distinct ways (Santulli, 2014). For example, in a healthy kidney, ANGPTL1 is weakly expressed; however, its role in the kidney disease has not been investigated (Lai et al., 2007).

ANGPTL2 is identified as a chronic inflammatory adipokine (Morinaga et al., 2016). Levels of ANGPTL2 are higher in individuals with obesity or kidney problems and are produced by tubular epithelial cells in the kidney (Huang et al., 2019; Li et al., 2013). Under stresses, such as hypoxia and diabetes, ANGPTL2 has shown its elevated expression (Huang et al., 2019). Morinaga et al. (2016) identified in mice that genetic deletion of ANGPTL2 reduces the chance of tubulointerstitial fibrosis and suppresses TGF-β1/Smad signaling, a major renal pathway that causes fibrosis and scarring (Morinaga et al., 2016). Usui et al. (2013) found, at the population level, that higher levels of ANGPTL2 in blood were associated with a greater prevalence of chronic kidney disease and albuminuria (Usui et al., 2013). This study was completed free of other major risk factors that could confound the association. Therefore, ANGPTL2 may serve as a biomarker and mediator of kidney inflammation and fibrosis during diabetes (Ishii et al., 2019).

ANGPTL3, which is a liver-secreted protein that is significantly elevated in kidney diseases, involving podocyte injury, proteinuria, and DKD (Tamehri Zadeh et al., 2025; Liu et al., 2015). Elevated ANGPTL3 levels promote lipid abnormalities and podocyte dysfunction, making it a potential biomarker of disease severity and a promising therapeutic target (Azadi et al., 2023; Wen et al., 2023; Ma et al., 2023).

The correlation data of plasma ANGPTL5 levels in the subjects with type II diabetes and obesity suggest the roles in lipid and glucose metabolism, obesity, and diabetes mellitus (Liu et al., 2021; Alghanim et al., 2019; Hammad et al., 2020). There is limited direct evidence of its specific function in kidney disease (Liu et al., 2021).

ANGPTL6 has shown a notable expression in kidney disease, particularly in the context of diabetes and renal clear cell carcinoma (Yang et al., 2023; Qaddoumi et al., 2020; Fan et al., 2020; Takebayashi et al., 2025). Genetic variants of ANGPTL6 have been identified as both potential risk factors and diagnostic biomarkers. The C allele of the *ANGPTL6* rs8112063 gene variant is linked with an increased risk of DKD, suggesting a significant role in the progression of kidney damage in patients with diabetes (Swiderska et al., 2022). In addition, ANGPTL6 is a liver-derived circulating molecule that plays a critical role in regulating metabolic homeostasis (Qaddoumi et al., 2020). In addition, ANGPTL6 functions as a proangiogenic factor, promoting the formation of new blood vessels; however, its role in renal angiogenesis remains unexplored (Okazaki et al., 2012). Genetic variants that lead to defective or reduced ANGPTL6 protein may contribute to abnormal vessel proliferation or structural weakness, which has been linked to conditions, such as familial intracranial aneurysms (Hostettler et al., 2021).

ANGPTL7 is a secreted protein that has roles in inflammation, angiogenesis, insulin resistance (Xu et al., 2020), and lipid metabolism through diverse mechanisms, such as reducing the protein levels of insulin receptor and insulin receptor substrate1; increasing the expression of suppressor of cytokine signaling 3 (SOCS3); promoting the degradation of IRS1 in the proteasome (Xu et al., 2020); inhibiting AKT phosphorylation and activation of ERK1/2 (Xu et al., 2020). ANGPTL7 is highly expressed in the eye, where it helps maintain corneal avascularity (lack of blood vessels) (Comes et al., 2011). Rare variants in the *ANGPTL7* gene are associated with lower intraocular pressure and a reduced risk of glaucoma (Comes et al., 2011).

ANGPTL8 is a regulator of lipid metabolism that acts as a novel biomarker and pathogenic mediator of CKD and DKD progression (Pan et al., 2026; AlMajed et al., 2023). Elevated serum and urine levels of ANGPTL8 strongly correlate with kidney dysfunctions, with some studies showing a 2.59-fold higher risk in individuals with high compared to low levels, elevated tubular inflammation, and renal fibrosis (Meng et al., 2021; Zou et al., 2021). ANGPTL8 levels are significantly increased in patients with type 2 diabetes and albuminuria (AlMajed et al., 2023). It is positively correlated with the urine albumin-to-creatinine ratio and inversely with the estimated glomerular filtration rate (AlMajed et al., 2023). The pathogenic mechanism underlying ANGPTL8-mediated kidney injury is likely to promote inflammation and fibrosis in renal tubular epithelial cells, possibly through interaction with the Akt2 pathway (Pan et al., 2026). Studies suggest that inhibiting ANGPTL8 may help protect renal tubular cells from injury, specifically in DKD (AlMajed et al., 2023). In addition, in malignant renal cell carcinoma, ANGPTL8 is induced by inflammatory stress and supports tumor growth by maintaining an undifferentiated cellular state (Matsukawa et al., 2023).

In contrast to ANGPTL1, which has shown protective roles in kidney injuries, the other broader ANGPTL protein family (ANGPTL2, ANGPTL3, ANGPTL4, and ANGPTL8) has established roles in various renal pathologies, including DKD, and the specific involvement of ANGPTL5 and ANGPTL6 in the pathogenesis or progression of primary kidney disease, or in DKD, is less understood.

## 3 The ANGPTL4 in renal metabolic health and disease

ANGPTL4 is highly expressed in the liver and adipose tissue and is strongly induced by fasting and hypoxia in these organs (Morelli et al., 2020; Aryal et al., 2019; Xu et al., 2005). It is a target gene of the nuclear receptors peroxisome proliferator-activated receptors (PPAR)-α and PPAR-γ (Liu et al., 2017; Kersten, 2021). ANGPTL4 is a potent inhibitor of lipoprotein lipase and induces marked hypertriglyceridemia after intravenous injection or adenovirus-mediated expression (Sylvers-Davie and Davies, 2021). Population-based studies of ANGPTL4 have identified variants that affect triglyceride levels (Romeo et al., 2007). Most normal circulating ANGPTL4 in rodents is secreted from the liver as a cleaved protein that binds high-density lipoprotein particles (Romeo et al., 2007). Within individuals with obesity and type 2 diabetes, Cinkajzlová et al. (2018) found that higher blood ANGPTL4 levels are associated with insulin resistance, increased triglycerides, and reduced HDL cholesterol (Cinkajzlova et al., 2018). These strong associations point to ANGPTL4’s value as a clinical biomarker, helping doctors identify people at risk of metabolic syndrome (Cinkajzlova et al., 2018). Recent research has shown that ANGPTL4 does not just have fat- and lipid-metabolic implications but also affects how cells use specific amino acids, particularly glutamine in cancer cells (Xiao et al., 2022) found that overexpression of ANGPTL4 in cancer cells increased glutamine absorption and upregulated FAO genes (Xiao et al., 2022). Together, these processes boost cellular ATP production, supporting cell survival even under stress. Collectively, these data demonstrate that ANGPTL4 is involved in multiple metabolic pathways, influencing glucose, lipid, and amino acid metabolism. It has several direct and indirect consequences for health and disease progression.

ANGPTL4 has been shown to play diverse roles in kidney health and disease (Li et al., 2024b; Chugh et al., 2014; Wang X. et al., 2024; Shen et al., 2025). ANGPTL4 is primarily produced in podocytes, a key component of the kidney’s glomerular filtration system, and in tubular epithelial cells (TECs) (Chugh et al., 2014; Shen et al., 2025; Jia et al., 2020). In healthy kidneys, ANGPTL4 regulates glomerular permeability and endothelial cell junctions, maintaining tight intercellular spaces (Srivastava et al., 2025a). The glomerular expression of ANGPTL4 is glucocorticoid sensitive and is highly upregulated in the serum and in podocytes in experimental animal models of MCD and in human MCD (Clement et al., 2011). Podocyte-specific transgenic overexpression of ANGPTL4 in rats induced nephrotic-range and selective proteinuria, loss of glomerular basement membrane charge, and foot process effacement, whereas transgenic expression specifically in the adipose tissue resulted in increased circulating ANGPTL4, but no proteinuria (Clement et al., 2011). ANGPTL4 secreted from podocytes in some forms of nephrotic syndrome lacks normal sialylation (Clement et al., 2011). When fed the sialic acid precursor N-acetyl-D-mannosamine to ANGPTL4-overexpressing transgenic rats, it increased ANGPTL4 sialylation and decreased albuminuria (Clement et al., 2011). The cumulative effects of these results suggest that podocyte-secreted ANGPTL4 plays a key role in nephrotic syndrome (Clement et al., 2011). In addition, an increase in circulating ANGPTL4 levels (presumably the normo-sialylated form, based on its neutral isoelectric point in adipose tissue) in response to nephrotic-range proteinuria reduces the severity of this pathology, but at the cost of inducing hypertriglyceridemia, while also suggesting a possible therapy to treat these linked pathologies (Jia et al., 2020; Del Nogal-Avila et al., 2016).

## 4 The role of ANGPTL4 in diabetic kidney disease

Human studies have shown a positive correlation between ANGPTL4 levels and expression of characteristics of diabetic nephropathy (Abedini et al., 2024). In cultured glomerular mesangial cells, ANGPTL4 deficiency inhibits both the inflammatory response and extracellular matrix accumulation (Qin et al., 2019). In diabetic conditions, ANGPTL4 is markedly upregulated in podocytes and tubular cells, leading to several pathological changes (Srivastava et al., 2024) used genetically engineered mouse models to demonstrate that deleting ANGPTL4 in podocytes and/or tubular cells can prevent proteinuria, glomerular and interstitial fibrosis, and preserve kidney function in diabetic mice (Srivastava et al., 2024). Kidney biopsies from humans with DKD reveal that higher levels of ANGPTL4 led to more albuminuria, an essential marker of kidney damage, and more severe histological injury (Pan et al., 2026). ANGPTL4’s influence extends to many other pathways and mechanisms, with system-wide effects. It amplifies the TGF-β/Smad and NF-κB signaling pathways, especially in the podocyte and tubular regions of the kidney (Srivastava et al., 2024). The result is increased collagen and fibronectin production, leading to increased inflammation in kidney tissues (Li et al., 2024a; Srivastava et al., 2024). ANGPTL4 can further affect podocyte architecture: increased levels correlate with a higher risk of foot process effacement and alterations in slit diaphragm proteins, both of which contribute to albuminuria (Li et al., 2024a). Recent clinical evidence suggests that ANGPTL4 is a biomarker for severe DKD and kidney function loss; blood and urinary ANGPTL4 levels are higher in individuals with more severe DKD, and these levels correlate with disease severity and kidney function loss (Wang Y. et al., 2024). Altogether, ANGPTL4 has implications for metabolism, inflammation, and microvascular injury, ultimately worsening DKD (Li et al., 2024a).

Hypoxia-inducible Factor (HIF) transcriptionally upregulates ANGPTL4 expression in diabetic kidney tissues (Li et al., 2024b). Another transcription factor that regulates ANGPTL4 during hypoxia is Signal transducer and activator of transcription 3 (STAT3), which is commonly activated by inflammatory cytokines, and enhances ANGPTL4 transcription when paired with HIF signaling (Inoue et al., 2014; Chong et al., 2014; Dinarello et al., 2023). In addition, ANGPTL4 is a well-established transcriptional target of PPARα, PPARβ, and PPARγ (Georgiadi et al., 2010). Activation of PPAR pathways, resulting from hypoxia-induced alterations in lipid metabolism, further upregulates ANGPTL4 expression. Furthermore, Retinoic acid receptor-related orphan receptor alpha (RORα) binds to regulatory elements within the ANGPTL4 promoter to promote transcription, while MYC proto-oncogene protein (c-Myc) similarly enhances the transcription by modulating metabolic pathways (La et al., 2017).

Upregulation of ANGPTL4 typically results from hypoxic conditions, which enhance the profibrotic environment and drive the progression of renal interstitial fibrosis in DKD (Srivastava et al., 2024; Shen et al., 2025). It has also been reported that elevated ANGPTL4 expression has been associated with increased activation of the transforming growth factor-beta (TGF-β) pathway, a principal signaling cascade responsible for kidney fibrosis in DKD (Srivastava et al., 2024). This activation leads to excessive extracellular matrix production and promotes fibroblast activation.

Although ANGPTL4 does not directly activate TGF-β receptors, it significantly amplifies cellular profibrotic signaling through mechanisms such as activating the dipeptidyl peptidase-4 (DPP-4) pathway and upregulating pro-inflammatory cytokines (Srivastava et al., 2024). Furthermore, stimulation of the TGF-β receptor complex initiates intracellular signaling that triggers phosphorylation of SMAD2 and SMAD3, which, together with SMAD4, form an active transcription complex that binds to the regulatory element of the ANGPTL4 promoter (Srivastava et al., 2024; Li et al., 2017). This process establishes a positive feedback loop in which ANGPTL4 sustains profibrotic signaling through TGF-β, while TGF-β signaling further upregulates *ANGPTL4* gene expression (Srivastava et al., 2024; Li et al., 2024b; Shen et al., 2025). Collectively, these findings indicate that hypoxia-induced HIF-1α upregulates ANGPTL4, which directly amplifies TGF-β–mediated fibrotic signaling via SMAD-dependent transcription factors. This integrated model suggests that inhibiting ANGPTL4 may be a promising strategy to slow the progression of DKD.

### 4.1 The role of ANGPTL4 in fibrotic vs. nonfibrotic diabetic kidney

ANGPTL4 is a known inhibitor of LPL, an enzyme that catalyzes the hydrolysis of triglycerides into fatty acids, which are used by peripheral tissues and kidney tubules (Kersten, 2021; Wang X. et al., 2024; Lafferty et al., 2013; Li Y. et al., 2022). Upregulated ANGPTL4 expression and suppressed LPL expression, along with their associated activities, are key fibrogenic factors in the mouse model of DKD (Bhatia and Srivastava, 2025; Srivastava et al., 2025a; Srivastava et al., 2024). Analysis of diabetic kidneys from global ANGPTL4 mutant mice and podocyte- and tubule-specific ANGPTL4 mutant mice showed suppressed levels of renal fibrosis, proteinuria, proinflammatory cytokines, and mesenchymal activation in tubules and endothelial cells, suggesting that renal ANGPTL4 is associated with fibrosis, proteinuria, inflammation, and mesenchymal activation in diabetic kidneys (Srivastava et al., 2024). ANGPTL4 is a catalyst of renal fibrosis in diabetes and disrupts cytokine and chemokine reprogramming by upregulating TGFβ signaling (Srivastava et al., 2024). These processes alter metabolic homeostasis by predisposing toward defective fatty acid metabolism and associated mesenchymal activation in tubules and endothelial cells (Srivastava et al., 2024). Of note, the CD-1; db/db mouse, a type 2 diabetic mouse model that mimics the renal fibrotic features of human DKD, has significantly higher levels of renal ANGPTL4 expression, suggesting a potential role for ANGPTL4 in DKD (Mizunuma et al., 2020).

### 4.2 The role of ANGPTL4 in mesenchymal metabolic shifts in diabetic kidney

Defective metabolism, the gain of mesenchymal marker proteins in diverse kidney cell types, immune cell activation, proinflammatory cytokines, aberrant chemokine levels, and tubular cell apoptosis are key phenomena driving fibrosis in the diabetic kidney (Thomas et al., 2015; Srivastava and Kanasaki, 2022; Srivastava et al., 2020a; Li et al., 2020a; Srivastava et al., 2018; Srivastava et al., 2021c; Abedini et al., 2024; Kang et al., 2015; Srivastava et al., 2025b). A study on spatial transcriptomics in the human kidney identified four distinct cellular microenvironments: glomerular, immune, tubule, and fibrotic, and provided a comprehensive molecular roadmap of the human kidney and the fibrotic process, demonstrating the importance of cell specificity in the development of fibrosis (Abedini et al., 2024). Myofibroblasts can originate from epithelial cells via partial epithelial-to-mesenchymal transition (EMT), from endothelial cells via endothelial-to-mesenchymal transition (EndMT), from macrophages via macrophage-to-mesenchymal transition, and from resident fibrocytes from bone marrow origin (Li et al., 2020a; Srivastava et al., 2021b; Srivastava et al., 2021c; Srivastava et al., 2013; Srivastava et al., 2021d; Srivastava et al., 2019; Huang and Susztak, 2016; Lovisa et al., 2015; He et al., 2025; Yang et al., 2010; Allison, 2015; Sugimoto et al., 2012; Lovisa et al., 2020; Zeisberg et al., 2008; Li et al., 2020b). These myofibroblasts express mesenchymal markers (α-smooth muscle actin, N-cadherin, and vimentin) and contribute to organ fibrogenesis. Intermediate cell types that arise during mesenchymal activation can activate mesenchymal activation in healthy neighboring renal cells (Srivastava et al., 2021c; Huang and Susztak, 2016; Lovisa et al., 2015; He et al., 2025; Yang et al., 2010; Allison, 2015; Sugimoto et al., 2012; Lovisa et al., 2020; Zeisberg et al., 2008; Li et al., 2020b). Biological pathways such as activated transforming growth factor-β (TGFβ) signaling, Notch signaling, Wnt signaling, and Hedgehog signaling disrupt central metabolism and drive mesenchymal metabolic shifts in diabetic kidneys, ultimately leading to deposition of collagens, extracellular matrix proteins, fibronectin, vimentin, and N-cadherin in interstitial spaces (Huang and Susztak, 2016; Lovisa et al., 2015; He et al., 2025; Yang et al., 2010; Allison, 2015; Sugimoto et al., 2012; Lovisa et al., 2020; Zeisberg et al., 2008; Li et al., 2020b).

To begin to understand how ANGPTL4 accelerates mesenchymal activation in diabetic kidneys, ANGPTL4 has a prominent effect on *de novo* lipogenesis (DNL) (Srivastava et al., 2024). In diabetic kidneys with fibrosis, elevated TG levels (lipid load) arising from defective mitochondrial lipid peroxidation are not observed in ANGPTL4 mutant mice (tubule-specific or podocyte-specific) (Srivastava et al., 2024). In addition, ANGPTL4 loss improved the mitochondrial structure and function and restored lipid oxidation in diabetes (Srivastava et al., 2024). Increased lipid oxidation has been observed in tubules that overexpress Cpt1a, which have demonstrated enhanced mitochondrial homeostasis and reduced renal fibrosis in mice (Miguel et al., 2021). Lipid accumulation and expression of genes related to DNL, along with the concomitant induction of aberrant glycolysis, are elevated in the kidneys of patients with fibrosis (Mukhi et al., 2023) and in a mouse model of diabetes. Moreover, Acyl-CoA synthetase short-chain family 2 regulates DNL, leading to NADPH depletion and increased ROS levels, ultimately triggering NLRP3-dependent pyroptosis in kidney tubules (Mukhi et al., 2023). Dysregulated lipid accumulation, defective fatty acid oxidation, and elevated glycolysis-lactate pathway activity lead to mitochondrial damage, and these processes are linked to myofibroblast metabolic shifts in diabetic kidneys (Kang et al., 2015; Mukhi et al., 2023). Therefore, mitigating ANGPTL4 levels in renal cell types is a crucial step in the anti-mesenchymal and antifibrotic mechanisms that occur in kidney tubules and endothelium during diabetes.

### 4.3 The role of ANGPTL4 in regulating c-GAS-STING pathways in diabetic kidney

Defective central metabolism and related mitochondrial damage are critical in the development of kidney disease in diabetes (Srivastava et al., 2021c; Srivastava et al., 2025a; Srivastava et al., 2024; Srivastava et al., 2020c). These abnormal metabolic processes lead to cellular energy deficits, compromised organ function, and mitochondrial dysfunction, which, in turn, are associated with the release of mitochondrial contents into the cytosol, where they are recognized as “foreign” particles. This recognition inappropriately activates the immune system, triggering pathological inflammation via the cytosolic c-GAS-STING sensing pathway, leading to aberrant cytokine expression and proinflammatory immune cell recruitment in diabetes (Chung et al., 2019; Riley and Tait, 2020). Blocking this cytosolic c-GAS-STING sensing pathway reduces the phenotype of DKD and offers an unexplored therapeutic approach for patients with chronic kidney disease (Chung et al., 2019; Zang et al., 2022). ANGPTL4-deficient diabetic podocytes and tubules showed reduced inflammation, decreased mitochondrial component release, decreased c-GAS-STING sensing activation, and lower levels of proinflammatory cytokine expression, suggesting that ANGPTL4 deficiency can restore mitochondrial homeostasis and suppress diabetes-associated renal inflammation, both of which are critical phenotypes in DKD (Srivastava et al., 2024).

Macrophage polarization defined as the conversion of M2-type macrophages to pro-inflammatory M1-type macrophages, represents a crucial phenotype in the DKD. This process accelerates mesenchymal activity in both macrophages and adjacent tubular cells, thereby promoting fibrogenesis in the diabetic kidney (Youssef et al., 2024; Li HD. et al., 2022). Elevated levels of ANGPTL4 and c-GAS-STING pathways are associated with macrophage polarization and accumulation in the diabetic kidney (Srivastava et al., 2024; Li HD. et al., 2022). Previous studies, using a mouse model of obstruction (UUO), has shown that cGAS–STING signaling was activated in the kidneys during the development of renal fibrosis (Jiao et al., 2025). Mice lacking cGAS or STING exhibited significantly suppressed levels of macrophage proinflammatory activation, myofibroblast formation, and ECM protein production in the kidneys (Jiao et al., 2025). Pharmacological inhibition of cGAS using RU.521 reduces proinflammatory macrophage activation, myofibroblast formation, and renal fibrosis in the UUO mouse model (Jiao et al., 2025). The cGAS–STING pathway in macrophages is activated by double-stranded DNA released from damaged tubular epithelial cells, which subsequently promotes inflammatory responses (Jiao et al., 2025). STAT6 serves as a critical regulator of M2 macrophage polarization and is closely linked to PPARγ signaling. It is essential for the transcription of ANGPTL4 and fatty acid binding protein 4 (FABP4) (Szanto et al., 2010). STAT6 drives ANGPTL4 gene transcription and is responsible for macrophage polarization in both diabetic and nondiabetic chronic kidney diseases (Jiao et al., 2021a; Liu et al., 2023; Jiao et al., 2021b; Naik et al., 2026).

The STAT6-ANGPTL4 axis may be directly or indirectly associated with activation of the cGAS-STING pathway, macrophage polarization, and macrophage-to-mesenchymal transition in the diabetic kidney, thereby promoting renal fibrosis.

### 4.4 Podocyte- and tubule-secreted ANGPTL4 activate pro-mesenchymal dipeptidyl peptidase-4-integrin β1 transduction pathway in diabetic kidney

Active TGF-β signaling and higher dipeptidyl peptidase-4 (DPP-4) levels are causative pathways that accelerate renal fibrosis in diabetes (Srivastava et al., 2020b; Kanasaki et al., 2014). DPP-4-Integrin-β1 signaling levels and the associated TGFβ dimerization, which is the first step in activating the TGFβ signaling transduction cascade (Shi et al., 2015). Activated TGFβ signaling is one mechanism that suppresses lipid oxidation and induces the glycolysis-lactate pathways, thereby promoting “mesenchymal metabolic shifts” and related fibrogenesis in renal tubules (Srivastava et al., 2018; Kang et al., 2015). DPP-4 is a molecule that accelerates TGF-β-associated mesenchymal activations and related fibrosis in diabetic kidney (Kanasaki et al., 2014; Shi et al., 2015; Scheen, 2010). ANGPTL4 interacts with Integrin-β1 and activates the mesenchymal signal cascade in diabetic tubules and diabetic endothelial cells (Srivastava et al., 2025a; Srivastava et al., 2024). Podocyte-secreted ANGPTL4 activates the DPP-4-Integrin-β1 interactions and TGFβR1 and TGFβR2 dimerization, resulting in the activation of a cascade of signaling processes mediated through Smad proteins, primarily through Smad2/3 (Srivastava et al., 2024). Phosphorylated Smad3 alters the cytoplasmic pathway and enters the nucleus, where it represses the transcription of fatty acid oxidation genes and induces the transcription of genes related to aberrant glycolysis and fibrogenesis in tubules (Kang et al., 2015). In podocytes, augmentation of the DPP-4-Integrin β1 signaling cascade led to loss of GBM charge, alterations in podocyte cell permeability, foot process effacement, and podocyte cell death, and these cumulative effects lead to associated activation of mesenchymal signal transduction in tubules and endothelial cells, foot process effacement, increased albuminuria, and podocyte death (Srivastava et al., 2024). MicroRNA array data from the kidneys of nondiabetic mice and diabetic mice with severe renal fibrosis suggest miR-29-3p is critical in regulating ANGPTL4 expression levels by targeting the 3′UTR of its mRNA. Also, miR-29-3p targets the 3′UTR of DPP-4 mRNA and regulates mesenchymal-to-epithelial transdifferentiation and fibrogenesis (Kanasaki et al., 2014; Srivastava et al., 2024). miR-29b-3p deficiency in diabetic tubules is identified as a key regulator of DPP-4 and ANGPTL4 levels (Srivastava et al., 2024). Inhibition of miR-29 through LNA–miR-29 treatment has been shown to promote favorable plaque remodeling in atherosclerotic mice (Ulrich et al., 2016), worsen the features of fibrosis, distort kidney structure, and cause severe proteinuria in mouse models of DKD; in separate studies, miR-29b was reported to be reno-protective in db/db mice and STZ-induced diabetic mice (Nitta et al., 2016; Srivastava et al., 2021d; Chen et al., 2014; Srivastava et al., 2016; Nagai et al., 2014; Srivastava et al., 2014; Meng et al., 2021; Zou et al., 2021). These results suggest that miR-29-3p is a crucial regulator of both DPP-4 and ANGPTL4 mRNA and influences fibrogenesis in diabetic kidneys.

### 4.5 Therapeutic targeting of ANGPTL4 in diabetic kidney disease

ANGPTL4 plays a key role in DKD, making it a prime target for intervention with emerging therapies (Srivastava et al., 2021a; Bhatia et al., 2023). Preclinical research has tested techniques to block ANGPTL4 using ASOs, monoclonal antibodies, and small molecules (Gusarova et al., 2018; Cummings et al., 2025; Kirsch et al., 2017). Previous studies have demonstrated that ANGPTL4 is a potential metabolic regulator associated with many metabolic diseases (Dai et al., 2021; Spitler et al., 2021a; Spitler et al., 2021b). In one study, Dewey et al. used a neutralizing antibody of ANGPTL4 in both humanized mice and nonhuman primates, which resulted in severe side effects and systemic metabolic abnormalities (Dewey et al., 2016). Whole-body Angptl4 mutant mice and podocyte-specific and tubule-specific mutant mice suggest that ANGPTL4 has promising drug targetability against DKD (Srivastava et al., 2024). Therapies have already been developed to successfully inhibit ANGPTL3, which is now used to manage familial hypercholesterolemia and serves as a guide for developing drugs targeting ANGPTL4 (Luo et al., 2024; Schonck et al., 2024; Gaudet et al., 2017). Systemic blocking of ANGPTL4 poses many risks and is a challenge for new therapies (Dewey et al., 2016). Over-inhibiting ANGPTL4 will lead to over-activation of LPL, causing extremely low blood triglyceride levels and even pancreatitis (Jung et al., 2020). Simultaneously, it could increase vascular leak in sensitive tissues (Thorin et al., 2023; Vahdat-Lasemi et al., 2025). ANGPTL4 also plays essential roles in immune modulation, so blocking it could disrupt innate immune responses and cause other unexpected side effects (Wee et al., 2022; Lichtenstein et al., 2010; Oteng et al., 2019).

siRNA-mediated knockdown of ANGPTL4 is significant because it inhibits ANGPTL4 expression in all tissues, potentially affecting the physiology and pathology of other organs, particularly the liver and adipose tissue. Consequently, the development of kidney-specific antisense oligonucleotide (ASO) or adeno-associated viral (AAV) transduction methods is advantageous, as these approaches can selectively inhibit ANGPTL4 in proximal tubules, glomerular podocytes, and glomerular endothelial cells, thereby avoiding the adverse systemic effects associated with whole-body ANGPTL4 loss (Srivastava et al., 2024).

**FIGURE 1:**
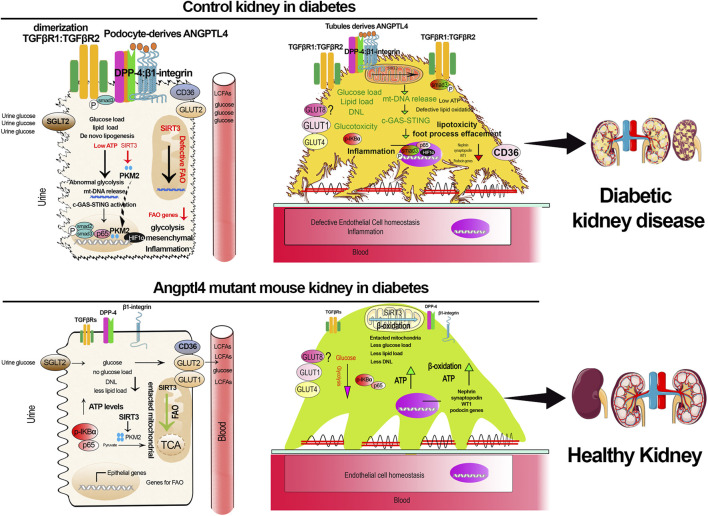
ANGPTL4 is a key fibrogenic molecule in diabetic kidney disease. The components in the figure was created using Servier Medical Art illustration resources.

In studies, kidney-specific ANGPTL4-ASO-treated mice exhibited partially restored kidney structure, substantially reduced glomerular and cortical fibrosis, and decreased proteinuria, without changes in body weight, blood glucose, or blood pressure in diabetic mice. Furthermore, no significant damage was observed in other tissues, such as the liver, adipose tissue, or heart (Srivastava et al., 2024). In summary, podocyte- and tubule-specific ANGPTL4 functions as a key profibrogenic molecule, and its depletion confers protection against DKD through metabolic reprogramming mediated by suppression of DPP-4-β1-Integrin and related TGFβ-smad3 signaling (Figure 1).

Upregulated ANGPTL4, DPP-4-integrinb1, lipid load, damaged mitochondria, and activated c-GAS-STING pathways led to higher inflammation and fibrogenesis in the diabetic kidney, whereas ANGPTL4 depletion demolished this fibrotic phenotype in the kidney during diabetes.

## 5 Future directions and clinical significance

Overall, ANGPTL4 demonstrates dual roles, serving as both a DKD biomarker and a therapeutic target (Srivastava et al., 2024; Wang Y. et al., 2024; Bano et al., 2023). Both clinical and animal studies support that blood and urine ANGPTL4 levels increase with DKD progression, associated with greater proteinuria and lower eGFR (Li et al., 2024a; Wang Y. et al., 2024). ANGPTL4 has implications beyond the kidney, including in diabetic eye disease, cancer, sepsis, and cardiovascular risk management, all of which highlight its systemic importance (Thorin et al., 2023; Li et al., 2024b; Qin et al., 2022; Ziveri et al., 2024; Ramos et al., 2025).

Several critical questions regarding ANGPTL4 remain unresolved, necessitating further research to facilitate the development of novel therapies. For example, strategies are needed to inhibit ANGPTL4 specifically in the kidney while minimizing adverse effects in other tissues. The fibrotic phenotype in kidney-specific ASO-treated mice should be analyzed in mouse models of type 2 diabetes. Future studies involve research into nanorobots and nanodelivery systems for targeting ANGPTL4, which may be used as a novel therapeutic option for metabolic disorders, such as hyperlipidemia, cancer, and atherosclerosis, as well as for inhibiting diabetic kidney fibrosis (Hu et al., 2024; Lokhande et al., 2025; Xiao et al., 2026; Wang et al., 2019). Bioengineering of targeted extracellular vesicles containing anti-ANGPTL4 nano molecules is an alternative approach to block elevated ANGPTL4 expression in the diverse cell types in diabetic kidney (Srivastava et al., 2026). While fully autonomous “robots” are still in the experimental stage, targeted nanocarriers and neutralizing antibodies are actively used, to modulate this protein’s effect on lipid metabolism (Hu et al., 2024; Lokhande et al., 2025). Using DNA Nanobots as programmable machines that alter their shape to create specific channels in plasma membranes, potentially allowing for the precise release of ANGPTL4-modulating proteins (Hu et al., 2024; Lokhande et al., 2025; Zhou et al., 2025; Chen et al., 2021). The development of Magnetic micro/nanorobots (MNRs) that are controlled by external magnetic fields to navigate blood vessels is another option for enabling targeted delivery of therapeutics (Hu et al., 2024; Lokhande et al., 2025; Wang et al., 2020; Zhou et al., 2021; Dai et al., 2026).

These devices may enable the targeted delivery of lipid-lowering agents or anti-ANGPTL4 molecules directly to kidney cells, thereby regulating impaired lipid metabolism and associated pathologies in diabetic kidneys (Wang et al., 2019; Paul et al., 2023; Lin et al., 2022). Enzyme-powered nanorobots, including advanced Janus nanorobots utilizing enzymes such as urease for propulsion, are capable of detecting chemical gradients to localize diseased tissue (Hu et al., 2024; Lokhande et al., 2025; Xu et al., 2021; Zhao et al., 2025; Zhang et al., 2023).

Currently, this technology is being investigated for precision cancer therapy, where ANGPTL4 often serves as a biomarker. However, future research should also evaluate its potential in diabetes and related renal complications (Lokhande et al., 2025; Paul et al., 2023; Lin et al., 2022). Notably, the long-term consequences of ANGPTL4 inhibition on other organs remain to be elucidated. Furthermore, the utility of ANGPTL4 as a biomarker for early diagnosis of DKD and for personalized therapeutic strategies warrants further investigation. Addressing these challenges will require an integrated approach that incorporates molecular biology, drug delivery systems, biomarker development, and clinical trials.

The ANGPTL family of proteins represents a promising and multifaceted area of research, although significant knowledge gaps remain. Investigating ANGPTL4 may provide valuable insights for the diagnosis and treatment of diabetic kidney disease. Continued ANGPTL research could facilitate the development of therapies to prevent rapid progression to chronic kidney disease in a substantial patient population.
